# Re-irradiation of multiple brain metastases using CyberKnife stereotactic radiotherapy: Case report

**DOI:** 10.1097/MD.0000000000027543

**Published:** 2021-10-15

**Authors:** Han Zhou, Tiancong Wu, Xixu Zhu, Yikun Li

**Affiliations:** a School of Electronic Science and Engineering, Nanjing University, Nanjing, China; b Department of Radiation Oncology The Fourth Affiliated Hospital of Nanjing Medical University, Nanjing, Jiangsu, China; c Department of Radiation Oncology, Jinling hospital, Jiangsu, China.

**Keywords:** case report, CyberKnife, multiple brain metastases, Re-irradiation, stereotactic radiosurgery

## Abstract

**Introduction:**

Brain metastasis (BM) is the commonest adult intracranial malignancy and many patients with brain metastases require two course radiotherapy. Re-irradiation is frequently performed in Radiotherapy (RT) departments for multiple brain metastases.

**Patient concerns:**

We present a case of a 55-year-old male patient suffering from brain metastases, who had previously received whole-brain radiotherapy (WBRT) and first CyberKnife Stereotactic Radiotherapy (CKSRT) for metastases, presented with a recurrence of metastasis and new lesions in the brain.

**Diagnoses:**

An enhanced computed tomography (CT) scan of the brain revealed abnormalities with double-dosing of intravenous contrast that identified >10 lesions scattered in the whole brain.

**Interventions:**

Re-irradiation was performed using CKSRT. The patient was treated with 30 Gy in 5 fractions for new lesions and 25 Gy in 5 fractions for lesion that were locally recurrent and close to brainstem lesions.

**Outcome:**

The lesions were well-controlled, and the headache of the patient was significantly relieved one month after radiotherapy. The total survival time of the patients was 17 months from the beginning of the Cyberknife treatment.

**Conclusion:**

The present case report demonstrates that CyberKnife therapy plays a significant role in the repeated radiotherapy for multiple metastatic brain tumors. CKSRT can be used as a salvage method in recurrent multiple brain metastases.

## 1 Introduction

Brain metastasis (BM) is the commonest adult intracranial malignancy, develops in 10% to 40% of patients with advanced-stage cancer, including lung, breast, melanoma, renal cell, and colorectal cancers.^[1]^ Although BMs are common, the clinical presentation varies depending on size, number, and location.^[2]^ Considering the survival rate of patients with multiple brain metastases is low, present treatment options include neurosurgical resection, whole-brain radiotherapy (WBRT), stereotactic radiosurgery (SRS) or radiotherapy, chemotherapy, or their combination.^[3,4]^ Multiple stereotactic platforms are available, including GammaKnife (GK) radiosurgery, linear accelerator-based volumetric modulated arc therapy, TomoTherapy (Accuray Inc, Sunnyvale, CA), and CyberKnife (Accuray Inc, Sunnyvale, CA).^[5,6]^Table 1 lists the various treatment methods for brain metastases.

**Table 1:** Retrospective analysis of the different treatment methods for brain metastasis patients.

Author	Year	No .of patient	No. of lesion	Median volume per tumor, cc	prescription dose ,Gy	SRS platform	Median OS	Treatment course
Nishizaki et al^[7]^	2006	71	148	2.9	The median dose is 20.7Gy	cyberknife	56 wk	First course
Wendy et Al^[8]^	2008	62	145	1.47	The mean prescribed dose was 20 Gy (range, 14–24 Gy)	cyberknife	8.3 mo	First course
Molenaar et al ^[9]^	2009	86	150	5.1	12–25 (Median 21)	Linear Acelerator	6.2 mo	First course
Chen et al^[10]^	2012	44	-----	----	20–30 Gy/1f/1d, 24–50 Gy/5–12 Gy/2–7f	Linear Accelerator	13.5 mo	First course
Skeie et al^[11]^	2013	80	140	6.13	The prescription dose was 21.1 (10–25.1) Gy	Gamma Knife	6 mo	First course
Tamari et al ^[12]^	2015	67	109	0.9 for SRS and 6.1 for SRT	24–30Gy/1–3f	cyberknife	13.1 mo	First course
Croker et al^[13]^	2015	61	107	5.76	Median dose of 24	Linear Accelerators	21 mo	Second course
Murovic et al^[14]^	2017	150	—	—	—	cyberknife	13 mo	First course
de le pena et al^[15]^	2017	49	152	6.71	20–26	CyberKnife	15.5 mo	First course
Sayan et al^[16]^	2019	18	53	6.2	20 ± 4.9	CyberKnife	—	First course

SRS is the earliest stereotactic technology that is achieved by administering a relatively large, single dose fraction to precisely defined targets using multiple convergent beams. SRS combined with WBRT is more common in 2 to 4 metastases; there are no standard guidelines on the number of brain metastases that can be treated with SRS. Although multiple phase III trials have evaluated SRS in the treatment of 1 to 4 brain metastases, there are limited data regarding the role of SRS for a larger number of metastases. However, most of the patients present with multiple BMs, which is challenging to treat. Although local failure after SRS is typical, in the study of Sahgal et al^[17]^ the failure rate was 27% among the patients treated with SRS alone, indicating, about 27% of patients need repeated treatment. These options include resection, whole-brain radiation therapy, laser ablation, and repeat SRS. However, there is a lack of robust evidence investigating the best therapeutic approach in case of further intracranial progression after the first SRS course and their respective selection criteria.

Repeat SRS can damage the central nervous system (CNS), CNS tissues are known to be highly sensitive to dose per fraction, and even a small degree of fractionation can potentially reduce the risk of radiation necrosis.^[8,18]^ A potential approach to decrease the risk of radiation injury in the re-treatment setting is to use fractionated SRS, which is called Stereotactic radiotherapy (SRT). Using this approach, treatment fraction is typically delivered in 3 to 5 fraction treatments. SRS/SRT is performed with CyberKnife, which is an established modern noninvasive technology for intracranial and extracranial Radiosurgery.

In this article, we reported a case of lung cancer patient with multiple brain metastases treated by repeated Stereotactic Radiotherapy platform—CyberKnife.

## 2 Case report

A 55-year-old male was first treated in the department of Respiratory Medicine center, for a long-term cough and pain in the chest and back. Using x-ray, a mass was detected in the right lobe of the lung. Enhanced computed tomography (CT) scans confirmed the suspicion of lung cancer and identified multiple brain metastases clinically staged as T4 according to the AJCC (The American Joint Committee on Cancer, AJCC) (7th edition) lung cancer staging system.

The patient had received surgery as a primary treatment and the postoperative pathological findings showed invasive adenocarcinoma; immunohistochemistry showed CK7 (+), TTF-1 (+), NapsinA (+), P53 (+++.80%), P40(−), Ki67 (+,40%), PD1 (lymphocyte+), PDL1 (tumor cell +), Elastic fibers (+). After the surgery, 1 cycle of AP chemotherapy and traditional Chinese medicine treatment was performed. Subsequently the patient presented with dizziness and discomfort. Head MR revealed multiple intracranial metastases. WBRT with prescription 30Gy/10fx was initiated followed by 5 cycles of Bevacizumab 600 mg, d0 + pemetrexed 1.0 g d1 albumin taxol mg chemotherapy, and then 6 cycle of Atzumab 1200 mg immunotherapy.

**Figure 1:**
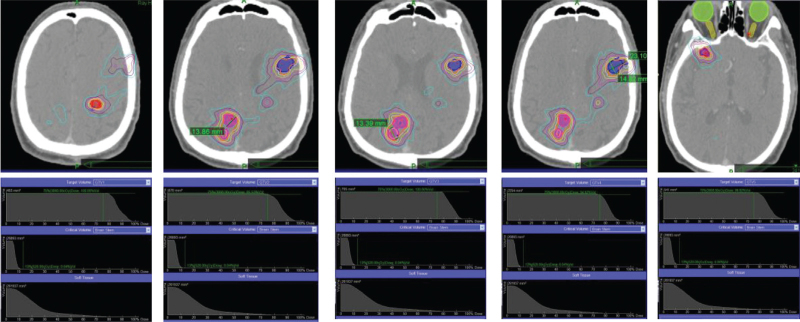
The location of brain tumors from positioning CT images and final excellent dose distribution for each tumor.

One year after the initial WBRT, the patient experienced severe headaches. An enhanced CT scan of the brain revealed abnormalities with a double-dose intravenous contrast, which demonstrated that there were no <5 lesions scattered in the whole brain, including the frontal, parietal and temporal lobes (Fig. 1). All BMs were located outside critical structures, and the patient was treated by CKSRT with prescription 35 Gy/5fx in the CyberKnife Radiotherapy center (summarized in in Table 2). Six months after the first SRT, magnetic resonance imaging (MRI) examination found that there were number of metastatic tumors, no <10 lesions scattered in the whole brain; The KPS of the patient was >80.

**Table 2:** The volume of each tumor for the first CyberKnife Radiotherapy of the case.

No.	Volume, cc	maximum size∗minimum size, mm
GTV_1_	0.403	8.5∗8.5
GTV_2_	0.87	8.5∗10.6
GTV_3_	0.705	13.11∗8.5
GTV_4_	2.254	31.4∗12.4
GTV_5_	0.54	8.2∗8.0

Considering the patient's survival time and the possible treatment, the patient was treated with re-irradiation using CyberKnife. All the tumors underwent repeated SRT using CyberKnife. The patient was in the supine position and fitted with a thermoplastic mask for immobilization. A high-resolution CT was obtained, followed by magnetic resonance imaging fusion imaging for target identification. The planning target volume (PTV) was generated by adding a margin of 1 mm to the gross tumor volume (GTV). The organs at risk, including the eyes, lenses, optic nerves, optic chiasm, brainstem and spinal cord, body (normal brain minus planning target volume) were delineated.^[19]^ Dose planning was performed with Multiplan Software (Accuray Inc, Sunyvale, CA).

Thirteen metastatic tumors were treated by the repeated CKSRT, median tumor volume was 0.463 cc ranging from 0.264 to 0.751cc, as shown in Table 3. The prescription dose and fractionation were decided as 30Gy/5fraction and 25 Gy/5fraction following the RTOG 9005 guidelines and the treating physician's preference according to radio-sensitivity of the primary tumor, tumor volume, tumor location that was located near critical structures, and prior doses received. The doses were planned according to volume.^[20]^ The treatment volumes were prescribed to 75% isodose line.

**Table 3:** The volume of each tumor for the irradiation with CyberKnife of the case.

No.	Volume, cc	Maximum size∗minimum size, mm	No.	Volume, cc	Maximum size∗minimum size, mm
GTV_1_	0.361	9.5∗5.4	GTV_8_	0.751	14.5∗13
GTV_2_	0.565	10.8∗4.2	GTV_9_	0.705	11.2∗11
GTV_3_	0.626	11.4∗8.21	GTV_10_	0.386	8.2∗8
GTV_4_	0.691	12.3∗10	GTV_11_	0.342	9∗9
GTV_5_	0.54	10.7∗7.9	GTV_12_	0.264	8.6∗7.6
GTV_6_	0.296	9.8∗9	GTV_13_	0.596	15.2∗7.5
GTV_7_	0.588	9.9∗9			

**Figure 2:**
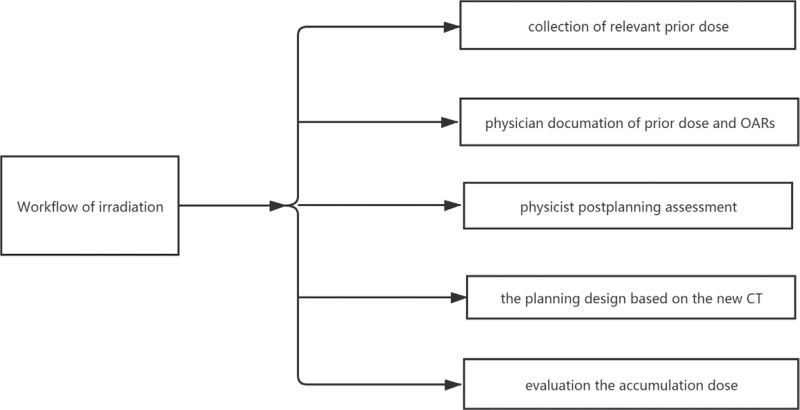
The workflow of re-radiation of the multiple brain metastases.

**Figure 3:**
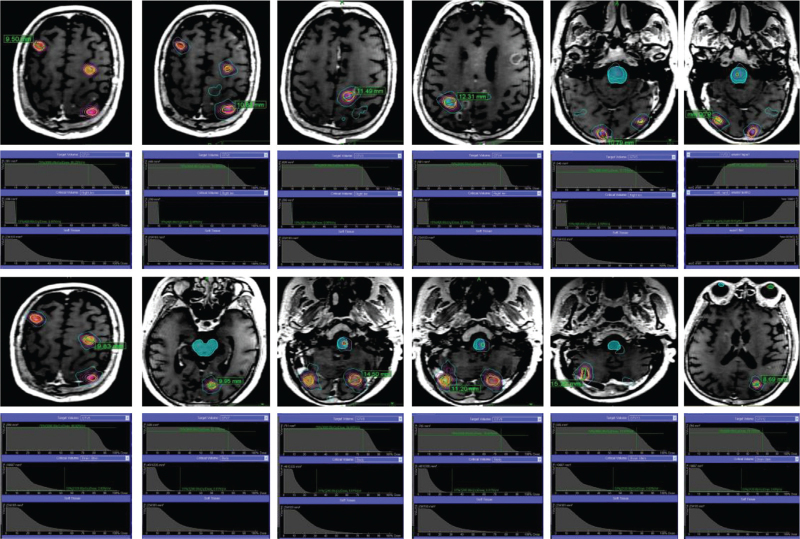
The location of tumors for the second cyberknife radiotherapy from positioning CT images and the excellent dose distribution of each tumor.

The prescription dose to the GTV and the organs at risk dose constraints were as follows: D90% of GTV received 100% of the prescription dose at least, for the optic nerve and chaism D0.2cc ≤ 23, for CyberKnife non-coplanar treatment. Dose constraints followed Task Group 101 recommendations for normal tissue. Reducing the toxicity was crucial due to the recurrent brain multiple brain metastases. Therefore, we designed the workflow of the re-irradiation^[21]^ (Fig. 2). The result of the repeated irradiation of the patient's brain multiple metastases was shown in Figure 3.

The treated lesions were well controlled, and the headache of the patient was significantly relieved 1 month after radiotherapy but for intracranial edema. The follow-up of the patient is 8 months after re-irradiation with CyberKnife therapy. The total survival time of the patient was 17 months from the beginning of the cyberknife treatment who died of systemic metastasis and multiple organ failure.

## 3 Discussion

Most patients with advanced lung cancer have BMs; the treatment for patients with BMs includes surgical resection, WBRT, repeat WBRT, SRS, and repeated SRS if the patient previously received SRS. As early as 1974 Shehata et al^[22]^ reported the use of repeat WBRT on patients with progressive BM, although WBRT has historically been the standard treatment for patients with multiple intracranial metastases, the addition of WBRT to SRS improves intracranial control, it has not been reported to translate into overall survival benefits and is associated with detrimental effects on neurocognitive function and a reduction in quality of life. However, there are major concerns about the administration of repeated SRS courses with regard to possible radiation overdose within the overlapping fields of multiple treatments and the uncertain impact on survival. The cumulative risk of radionecrosis of 5% to 12% has been reported after multi-fraction SRS with 27 to 35 Gy doses in 3 to 5 fractions.^[23]^ The prescription dose was selected on a case-by-case basis, depending on treatment volume, metastasis location and previous doses received. Moreover, some literatures recommended a dose of at least 24 Gy to achieve complete response.^[24]^

The CyberKnife described in 1997 is an image-guided frameless robotic technology for whole-body radiosurgery; it can be used for classic single-fraction radiosurgery and for hypofractionated treatments. The CyberKnife design coupled with real-time imaging have improved accurate target localization and dose delivery for brain tumors allowing higher biologically effective dose delivery without increased incidence of toxicity, which showed to be an effective technique for BMs.^[25]^ There is one case reporting the repeated SRS using Cyberknife, and the procedure of the repeated SRT was controlled well and also reduced the incidence of toxicity. From Table 3, we found when planning Stereotactic intracranial treatment, the emphasis should be on the total intracranial tumor volume involved rather than the number of lesions to be treated; the cumulative intracranial tumor volume of the present case was 11.129 cc, which is in well agreement with the previously published results.^[26]^ The principal advantage of CKSRT is the possible production of a peripheral dose falloff which reduces the amount of exposure of normal brain tissue. Unlike gamme knife and VMAT (Volumetric Intensity Modulated Arc Therapy, VMAT) radiotherapy,^[27]^ another advantage of CKSRT in our department is the procedure of the re-irradiation for the multiple brain metastases.^[28]^

From the perspective of tumor radiobiology, fractional radiotherapy is more consistent with the control principle of metastatic tumor. The high dose (15–25 Gy) radiotherapy of the lesion leads to a significant increase in the risk of radiation necrosis, especially when the tumor diameter exceeds 40 mm or it is close to an important structure.^[29]^ In addition, multiple brain metastases with more and larger lesions will increase the total treatment volume. Hence a single SRS treatment time will be prolonged, and the risk will increase accordingly. In this case, the use of hypofractionated stereotactic radiotherapy may be a better option; the treatment can not only reduce the risk of radiation necrosis but also maintain a better focus control effect.

## 4 Conclusion

Repeat CKSRT appears to be an effective treatment option for patients with brain metastases experiencing local failure following initial SRT treatment. This series demonstrates that repeat SRT may be indicated for selected cases of local disease recurrence.

As seen in this case report, the second course of SRT at doses of 25 to 30 Gy in 5 daily fractions appears to be a feasible treatment modality for progressive/recurrent brain metastases. It results in acceptable local control and risk of neurological toxicity. Further studies are needed to define the optimal management of patients with recurrent brain metastases using CyberKnife. Thus, the technique likely offers a more effective, noninvasive and frameless alternative to gamma knife or multi-isocentric SRS in managing multiple brain metastases.

## Author contributions

**Data curation:** Tiancong Wu, Yikun Li.

**Formal analysis:** Tiancong Wu.

**Investigation:** Yikun Li.

**Project administration:** Xixu Zhu.

**Supervision:** Xixu Zhu.

**Writing – original draft:** Zhou Han.

**Writing – review & editing:** Yikun Li.

## References

[1] Kotecha R Gondi V Ahluwalia MS Brastianos PK Mehta MP. Recent advances in managing brain metastasis. F1000research 2018. 10.12688/f1000research.15903.1 PMC6234720 30473769

[2] Pakneshan S Safarpour D Tavassoli F. Brain metastasis from ovarian cancer: a systematic review. J Neurooncol 2014;119:01–6. 10.1007/s11060-014-1447-9 24789253

[3] Koay E Sulman EP. Management of brain metastasis: past lessons, modern management, and future considerations. Curr Oncol Rep 2012;14:70–8. 22071681 10.1007/s11912-011-0205-9

[4] Rishi A Yu H. Current treatment of melanoma brain metastasis. Curr Treat Option Oncol 2020;21: 10.1007/s11864-020-00733-z 32350685

[5] Wang Y Wang E Pan L. A new strategy of CyberKnife treatment system based radiosurgery followed by early use of adjuvant bevacizumab treatment for brain metastasis with extensive cerebral edema. Journal of Neurooncol 2014;119:369–76. 10.1007/s11060-014-1488-0 24879376

[6] Potrebko PS Keller A All S. GammaKnife versus VMAT radiosurgery plan quality for many brain metastases. J Appl Clin Med Phys 2018;19: 10.1002/acm2.12471 PMC6236835 30288936

[7] Nishizaki T Saito K Jimi Y. The role of cyberknife radiosurgery/radiotherapy for brain metastases of multiple or large-size tumors. Minim Invasive Neurosurg 2006;49:203–9. 17041830 10.1055/s-2006-947998

[8] Wendy H Tran P Gordon L. Cyberknife for brain metastases of malignant melanoma and renal cell carcinoma. Neurosurgery 2009;64:A26-32. 19165071 10.1227/01.NEU.0000339118.55334.EA

[9] Molenaar R Wiggenraad R Verbeek-de Kanter A Walchenbach R Vecht C. Relationship between volume, dose and local control in stereotactic radiosurgery of brain metastasis. Br J Neurosurg 2009;23:170–8. 19306173 10.1080/02688690902755613

[10] Chen X Xiao JP Li XP. Fifty percent patients avoid whole brain radiotherapy: stereotactic radiotherapy for multiple brain metastases. a retrospective analysis of a single center. Clin Transl Oncol 2012;14:599–605. 22855144 10.1007/s12094-012-0849-4

[11] Skeie BS Enger P Christopher J. Gamma knife surgery of colorectal brain metastases: a high prescription dose of 25 Gy may improve growth control. World Neurosurg 2011;79:525–36. 10.1016/j.wneu.2011.09.019 22120263

[12] Tamari K Suzuki O Hashimoto N. Treatment outcomes using CyberKnife for brain metastases from lung cancer. J Radiat Res 2014;56:151–8. 25344929 10.1093/jrr/rru092 PMC4572587

[13] Croker J Chua B Anne Bernard A. Treatment of brain oligometastases with hypofractionated stereotactic radiotherapy utilising volumetric modulated arc therapy. Clin Exp Metastasis 2016;33:125–32. 26482476 10.1007/s10585-015-9762-x

[14] Murovic J Ding V Han SS Adler JR Chang SD. Impact of cyberknife radiosurgery on overall survival and various parameters of patients with 1–3 versus≥ 4 brain metastases. Cureus 2017;9:e1798. 29282442 10.7759/cureus.1798 PMC5741273

[15] de la Pena C Guajardo JH Gonzalez MF Gonzalez F Cruz B. CyberKnife stereotactic radiosurgery in brain metastases: a report from Latin America with literature review. Rep Pract Oncol Radiother 2018;23:161–7. 29760591 10.1016/j.rpor.2018.02.005 PMC5948325

[16] Sayan M Mustafayev TZ Sahin B. Evaluation of response to stereotactic radiosurgery in patients with radioresistant brain metastases[J]. Radiat Oncol J 2019;37: 10.3857/roj.2019.00409 PMC6952719 31918464

[17] Sahgal A Aoyama H Kocher M. Phase 3 trials of stereotactic radiosurgery with or without whole-brain radiation therapy for 1 to 4 brain metastases: individual patient data meta-analysis. Int J Radiat Oncol Biol Phys 2015;91:710–7. 25752382 10.1016/j.ijrobp.2014.10.024

[18] Bates JE Youn P Usuki KY. Repeat courses of SRS in patients initially treated with SRS alone for brain-metastatic melanoma. Melanoma Managt 2016;97–104. 10.2217/mmt-2016-0005 PMC6094646 30190878

[19] Shiohara S Ohara M itoh K. Successful treatment with stereotactic radiosurgery for brain metastases of endometrial carcinoma: a case report and review of the literature. Int J Gynecol Cancer 2003;13:71–6. 12631224 10.1046/j.1525-1438.2003.13017.x

[20] Buatti JM Friedman W Meeks S. RTOG 90–05: the real conclusion. Int J Radiat Oncol• Biol Phys 2000;47:269–71. 10802348 10.1016/s0360-3016(99)00506-4

[21] Paradis KC Mayo C Owen D. The special medical physics consult process for reirradiation patients. Adv Radiat Oncol 2019;4:559–65. 31681862 10.1016/j.adro.2019.05.007 PMC6817723

[22] Shehata WM hendricks FR Hind WA. Rapid fractionation technique and re-treatment of cerebral metastases by irradiation. Cancer 1974;54:257–61. 10.1002/1097-0142(197408)34:2<257::aid-cncr2820340206>3.0.co;2-6 4137158

[23] Inoue HK Seto Ken-ichi Nozaki A. Three-fraction CyberKnife radiotherapy for brain metastases in critical areas: referring to the risk evaluating radiation necrosis and the surrounding brain volumes circumscribed with a single dose equivalence of 14 Gy (V14). J Radiat Res 2013;4:04. 10.1093/jrr/rrt006 PMC3709677 23404206

[24] Shimamoto S Takehiro Inoue Hiroya S. CyberKnife stereotactic irradiation for metastatic brain tumors. Radiat Med 2002;20:299–304. 12553343

[25] Yang G Yishan W Wang Y. CyberKnife therapy of 24 multiple brain metastases from lung cancer: a case report. Oncol Lett 2013;6:534–6. 24137362 10.3892/ol.2013.1383 PMC3788854

[26] Michael A Moutrie V Rogers JM. Volume not number of metastases: Gamma Knife radiosurgery management of intracranial lesions from an Australian perspective. Radiother Oncol 2019;4:43–9. 10.1016/j.radonc.2018.12.018 30935580

[27] Sio TT Jang S Lee SW Curran B Pyakuryal SW Sternick ES. Comparing gamma knife and cyberknife in patients with brain metastases. J Appl Clin Med Phys 2014;15:14–26. 10.1120/jacmp.v15i1.4095 PMC5711245 24423830

[28] Juliette M Khalil T Dupic G. Second course of stereotactic radiosurgery for locally recurrent brain metastases: Safety and efficacy. PLoS One 2018;13:e0195608. 29621341 10.1371/journal.pone.0195608 PMC5886580

[29] Inoue HK Hiro S Seto K. Five-fraction CyberKnife radiotherapy for large brain metastases in critical areas: impact on the surrounding brain volumes circumscribed with a single dose equivalent of 14 Gy (V14) to avoid radiation necrosis. J Radiat Res 2014;55:334–42. 24187332 10.1093/jrr/rrt127 PMC3951086

